# Predictive value of prenatal ultrasound parameters for dichorionic growth discordant twins

**DOI:** 10.1186/s12884-020-03079-4

**Published:** 2020-07-29

**Authors:** Ting Yuan, Chao Li, Yan yan Wang, Wei Wang, Xue lan Li, Fen Li, Zhen Han

**Affiliations:** 1 Department of Obstetrics & Gynecology, the First Affiliated Hospital of Xi’an Jiaotong University, Xi’an, Shaanxi 710061 People’s Republic of China; 2 Department of Epidemiology and Biostatistics, School of Public Health, Xi’an Jiaotong University Health Science Center, Xi’an, Shaanxi 710061 People’s Republic of China

**Keywords:** Twin, Growth discordance, Prediction, Estimated fetal weight (EFW)

## Abstract

**Background:**

Twin growth discordance was demonstrated to be a risk factor for adverse perinatal outcomes, and prenatal ultrasonographic methods were utilized to predict twin growth discordance to improve outcomes. The results currently reported are not consistent due to the poor unified parameters and gestational durations.

**Methods:**

A total of 71 dichorionic twins with growth discordance and 346 dichorionic twins with normal growth were respectively included in the retrospective cohort study. The weight discordance of more than 25% was defined as a “growth discordant twin”. The clinical baseline information, maternal outcomes, twin birth weights and fetal growth parameters (which were measured by ultrasound) were compared between the two groups from early gestation to late gestation. Multiple logistic regression and receiver operating characteristic curves were adopted to evaluate the predictive value of the growth parameters.

**Results:**

Compared with normal dichorionic twins, the clinical baseline conditions were similar in the twins those finally developed growth discordance. The fetal growth parameters and the deepest volume pocket of amniotic fluid in early and mid-pregnancy showed no obvious predictive values. The fetal growth parameters in late pregnancy showed predictive values, especially the discordance of estimated fetal weight (EFW) in the early third-trimester (*P* < 0.001, area under the curve, 0.822; the cut-off value, 20%; sensitivity, 66.67%; specificity, 91.30%; positive predictive value, 88.46%).

**Conclusion:**

Two assessment approaches were suggested and adopted to predict twin growth discordance in the current study. Twin growth should be assessed longitudinally and dynamically. Normal twins may show growth imbalance in the early stage. The discordance of EFW in late pregnancy may be a useful indicator for a growth discordance of more than 25%, which is required further confirmation.

## Background

Twin pregnancy is a high-risk pregnancy, and adverse perinatal outcomes are likely to be complicated when growth discordance occurs. Growth discordance was demonstrated to be a risk factor for intrauterine death, especially when at least one fetus was small for gestational age (SGA) [1]; growth discordance also increased the risk of neonatal asphyxia [2] and affected the long-term neuro-development of the smaller twin [3]. Based on the adverse outcomes of growth discordance, much research has been carried out to investigate the ways to predict growth discordance and improve its outcomes. As in singletons, impaired twin growth should be assessed by comparing fetal biometry parameters against standards for uncomplicated pregnancy [4]. The predictive values of fetal growth parameters including biparietal diameter (BPD), head circumference (HC), abdominal circumference (AC), femur length (FL), and estimated fetal weight (EFW) were explored to a greater degree, but different or even opposite conclusions were reached [5, 6].

Overall, the research approaches predominantly compared the predictive values of some parameters during one gestational period; sometimes combined prediction evaluations were also performed. However, the results seemed to be disorderly and inconsistent due to the poor unified parameters and gestational durations [5–10]. In contrast, another approach was suggested. Because all fetal growth parameters could be obtained with regular ultrasound monitoring during the second trimester and the third trimester, we speculate that, the predictive value of some parameter (such as, EFW) in different gestational durations could be different, even for the same adverse outcome. This approach was performed to discover the optimal gestational duration in which the parameters have the best predictive values, thereby establishing better clinical significance and potential applications.

## Methods

### Enrolment

A total of 417 twin cases were retrospectively collected in the Department of Obstetrics and Gynecology in the First Affiliated Hospital of Xi’an Jiao tong University from March 2012 to December 2018. All of the cases had regularly examinations by prenatal ultrasound and finished deliveries in the department; the cases were all of dichorionic twins, and both twins survived. Cases of monochorionic twins, twins with unclear chorionicity, at least one twin death, and at least one twin with malformation or chromosomal abnormality were excluded. All chorionicity of twins were confirmed prenatally or postnatally according to guidelines [4]. 9 cases were excluded from discordant growth group and 17 cases were excluded from normal growth group.

The collected clinical information included maternal age, gravidity, parity, last menstruation period (LMP), pre-pregnancy weight, gestational weight change, height; maternal chronic diseases (including chronic hypertension, thyroid disease, chronic kidney disease, connective tissue disease, viral hepatitis, anemia, etc.), maternal obstetrical complications (including hypertensive disorder complicating pregnancy, pregnancy with diabetes, intrahepatic cholestasis of pregnancy, premature delivery, placenta previa, placental abruption, premature rupture of membranes, placental implantation, postpartum hemorrhage, etc.); and the birth weights of both surviving twins.

The following collected ultrasound data at the corresponding gestational age were: first trimester (11–13^+ 6^ weeks): crown-rump length (CRL), nuchal translucency (NT) and deepest volume pocket (DVP) of the amniotic fluid; second trimester (14–27^+ 6^ weeks): fetal biometric measurements (including BPD, HC, AC, FL) and DVP; and third trimester (28–38 weeks): fetal biometric measurements and DVP.

### Standardized manipulation of prenatal ultrasound scans [11]

Prenatal ultrasound was performed using a Voluson E8 color Doppler ultrasound system manufactured by GE company. With a convex array probe, the probe frequency was set at 3.5–5.0 MHz. When the CRL was between 45 and 84 mm, the NT measurement was performed by obtaining the median sagittal plane of the fetus. The image was enlarged and identified before the measurement. The fetus was occupied for more than 75% of the area on the image, and the height of the transparent layer between the skin and the fascia was vertically measured. The CRL was measured between the apex of the head and the lowest point of the buttocks in the median sagittal plane of the fetus. For the measurements of BPD and HC, the fetal thalamus plane was obtained, and the measurement of BPD was from the external edge of the skull aura to its internal edge, and the circumference measurement of the skull aura was obtained for the HC. For the AC measurement, the transverse plane of the abdomen was obtained to show the gastric vesicle on the left front side, the intrahepatic portal vein on the right front side and the transverse plane of the spine on the posterior side, and the measurement was included the skin layer. The FL was measured as the length of the femoral shaft. For the DVP measurement, we looked for the largest amniotic area that avoided the limbs, umbilical cord, etc., and the probe was held vertically in the measurement. In the study, the whole ultrasound scanning was performed by Han Z, who is the professor majoring at prenatal ultrasound for more than 20 years. Measurements were performed three times for each fetus and average values were used.

### Judgement criteria

To calculate the EFW, the Hadlock formula was used, as follows: Log_10_EFW = 1.5662–0.0108 × HC + 0.0468 × AC + 0.171 × FL + 0.00034 × HC^2^–0.003685 × AC × FL [7, 12]. “Weight discordance” was defined as [(larger twin weight – smaller twin weight) / larger twin weight] (%) [4], and the discordances of the other parameters were calculated as the same formula. A weight discordance of more than 25% was defined as a “growth discordant twin” [4].

### Research establishment and registration

The research was approved by the Ethics Committee of the First Affiliated Hospital of Xi’an Jiao tong University (Grant No. XJTU1AF2015LSL-073), and the clinical research project (NO. XJTU1AF-CRS-2015-003) has been established. Meanwhile, the project was also registered on the website of clinicaltrials.gov (ID: NCT02732717). The study procedure was compliant with STROBE guidelines.

### Statistical analyses

The data collected from the study subjects were verified and double entered into a data management system. The parameters were presented as the mean ± SD or as the median (min-max) and percentages, as appropriate. The statistical analyses were performed using the chi-square test, Fisher’s exact test, Student’s t-test, and Mann-Whitney U test, as appropriate. Multiple logistic regression was used to determine the odds ratio (OR) and 95% confidence interval (CI), and potential confounding factors were included in the analysis. The diagnostic values of the parameters were evaluated by the receiver operating characteristic (ROC) curve. All of the reported *P* values were 2-tailed, and values < 0.05 were considered statistically significant. The statistical analyses were performed using SPSS 16.0 (IBM, Armonk, NY, USA) and MedCalc 16.2 (MEDCALC, Ostend, Belgium).

## Results

### Baseline characteristics

There were no significant differences for the gestational ages on ultrasound for the first trimester, second trimester and early third trimester or for the other basic conditions between the two groups (*P* > 0.05). However, a significant difference was shown for the gestational age on ultrasound in the late third-trimester (*P* = 0.037) (Table 1).

**Table 1:** Comparison of baseline characteristics between growth discordant twin and normal twin

	Growth discordant group (***n*** = 71)	Normal growth group(***n*** = 346)	***P***
Maternal age ($\overline{x}\pm s$, yrs)	29.33 ± 4.72	29.42 ± 4.54	0.899
Pre-gestation BMI^a^ ($\overline{x}\pm s$, kg/m^2^)	22.56 ± 3.72	22.13 ± 1.50	0.157
Pre-delivery BMI($\overline{x}\pm s$, kg/m^2^)	28.15 ± 2.73	28.38 ± 3.53	0.923
Gravidity [M (min-max), times]	1 (0–3)	1 (0–6)	0.071
Nulliparity(n%)	48 (67.60)	259 (74.85)	0.207
Chronic diseases(n%)	9 (12.68)	55 (15.89)	0.493
Obstetrical complications(n%)	59 (83.10)	272 (78.61)	0.395
Week for ultrasound [M (min-max), w]			
First-trimester	13.00 (12.02–13.51)	13.00 (11.10–14.05)	0.221
Second-trimester	22.20 (17.02–26.03)	22.20 (21.05–27.61)	0.598
Early third-trimester	32.00 (28.03–33.00)	32.10 (28.05–34.05)	0.841
Late third-trimester	35.65 (34.01–40.30)	36.40 (34.12–40.00)	0.037
Delivery week [M (min-max), w]	36.52 (33.65–38.02)	37.10 (32.03–40.01)	0.347
Interval between delivery and the last ultrasound [M (min-max), d]	5.02 (3.15–8.12)	6.30 (4.05–8.19)	0.385

^a^*BMI* body mass index

### Comparisons of ultrasound parameters in the first trimester

The numerical values (the average value of twins, not shown in table) of CRL, NT and DVP were not significantly different between the two groups (*P* > 0.05). The discordances of CRL, NT and DVP were not significantly different between the two groups (P > 0.05) (Table 2).

**Table 2:** Comparisons of discordances of ultrasound parameters in the first-trimester between growth discordant twin and normal twin

Parameter^a^	Growth discordant group(***n*** = 71)	Normal growth group(*n* = 346)	***P***
CRL [M (min-max), %]	4.51 (1.56–5.33)	3.15 (0.00–22.22)	0.233
NT [M (min-max), %]	19.05 (5.26–23.81)	10.82 (0.00–49.17)	0.564
DVP [M (min-max), %]	6.51 (3.13–25.81)	10.31 (0.00–34.21)	0.866

^a^*CRL* crown-rump length; *NT* nuchal translucency; *DVP* deepest volume pocket

### Comparisons of the fetal growth parameters in the second and third trimester

The numerical values and discordances of BPD, HC, AC, FL, EFW and DVP were compared between the two groups for the three durations. In the second trimester, the comparisons of numerical values of all parameters showed no significances (*P* > 0.05). Only HC discordance was significantly greater in the case group than that in the control group (*P* < 0.05).

In the early third-trimester, the numerical values of BPD, HC, AC and FL were all significantly smaller in the case group than those in the control group (*P* = 0.025, 0.027, 0.015 and 0.036, respectively). The discordances of BPD, HC, AC, FL, and EFW were significantly greater in the case group than those in the control group (*P* < 0.05).

In the late third-trimester (for the twins who were examined for more than one time, the data at the last time was taken for analysis), the numerical values of AC, FL, and EFW were significantly smaller in the case group than those in the control group (*P* = 0.009, 0.008, and 0.030, respectively). The discordances of BPD, HC, AC, FL, and EFW were significantly greater in the case group than those in the control group (*P* < 0.05) (Table 3).

**Table 3:** Comparisons of discordances of ultrasound parameters in the second and third trimester between growth discordant twin and normal twin

Parameter^a^	Second-trimester			Early third-trimester			Late third-trimester		
	Growth discordant group(*n* = 71)	Normal growth group(*n* = 346)	***P***	Growth discordant group(*n* = 71)	Normal growth group(*n* = 346)	***P***	Growth discordant group(*n* = 71)	Normal growth group(*n* = 346)	***P***
BPD [M (min-max), %]	5.40 (0.30–13.16)	3.45 (0.00–10.91)	0.735	7.18 (0.00–17.98)	3.43 (0.00–12.94)	0.005	5.38 (0.00–11.46)	3.26 (0.00–12.94)	0.005
HC [M (min-max), %]	5.82 (0.17–13.48)	2.48 (0.00–10.45)	0.008	5.16 (0.00–17.97)	2.88 (0.00–9.28)	0.043	3.64 (0.33–11.29)	2.56 (0.00–12.57)	0.040
AC [M (min-max), %]	1.48 (0.00–15.08)	3.24 (0.00–14.98)	0.897	8.81 (1.26–25.90)	4.54 (0.00–15.09)	< 0.001	4.94 (0.68–26.90)	3.46 (0.30–20.56)	< 0.001
FL [M (min-max), %]	7.69 (2.03–20.83)	2.82 (0.00–7.89)	0.324	6.50 (1.61–22.95)	3.42 (0.00–12.73)	< 0.001	4.41 (0.00–14.71)	2.94 (0.00–12.50)	< 0.001
EFW [M (min-max), %]	11.06 (2.76–29.74)	10.87 (2.23–26.27)	0.713	21.32 (4.35–57.08)	12.38 (2.63–29.80)	< 0.001	16.75 (1.08–42.73)	10.56 (1.66–31.33)	< 0.001
DVP [M (min-max), %]	4.66 (0.00–27.50)	9.04 (0.00–72.97)	0.562	15.21 (2.50–57.53)	12.98 (0.00–59.52)	0.735	10.00 (0.00–61.36)	14.08 (0.00–76.09)	0.183

^a^*BPD* biparietal diameter; *HC* head circumference; *AC* abdominal circumference; *FL* femur length; *EFW* estimated fetal weight; *DVP* deepest volume pocket

### Associations between discordance of the fetal growth parameters in the second and third trimester with birth weight discordance

HC discordance in the second trimester was set as an independent variable in the multivariate logistic regression model, and growth discordance was set as the outcome variable, with adjustments for the second-trimester weeks for ultrasound and delivery week. The *P* value was 0.062, and the OR value was 1.37 [95% CI (0.98–1.90)].

The associations between the discordances of BPD, HC, AC, FL and EFW in the third trimester with birth weight discordance were respectively evaluated as the method described above. All *P* values were < 0.05 and detailed information were given (Table 4). These results were in accordance with those in Cox proportional hazards model, which were not presented here again.

**Table 4:** Associations between discordances of fetal growth parameters in the third-trimester with growth discordance by using multivariate logistic regression model^a^

Parameter	Early third-trimester						Late third-trimester					
	B	S.E.	Wald	***P***	OR	95%CI	B	S.E.	Wald	***P***	OR	95% CI
BPD	0.20	0.07	7.82	0.005	1.23	1.06–1.39	0.16	0.05	8.58	0.003	1.17	1.05–1.30
HC	0.28	0.10	7.92	0.005	1.32	1.09–1.61	0.17	0.07	6.66	0.010	1.19	1.04–1.35
AC	0.21	0.05	15.48	< 0.001	1.23	1.11–1.36	0.18	0.04	21.55	< 0.001	1.20	1.11–1.29
FL	0.27	0.06	17.84	< 0.001	1.31	1.16–1.49	0.32	0.05	34.79	< 0.001	1.38	1.24–1.53
EFW	0.16	0.04	19.81	< 0.001	1.17	1.09–1.26	0.16	0.03	38.23	< 0.001	1.17	1.11–1.23

^a^*BPD* biparietal diameter; *HC* head circumference; *AC* abdominal circumference; *FL* femur length; *EFW* estimated fetal weight; *OR* odds ratio; *CI* confidence interval

Note: relative risk is more suitable when prevalence of an outcome is more than 10%, despite common application and acceptance of OR

### Diagnostic values of discordances of the fetal growth parameters in the third trimester for growth discordance

The ROC curve was obtained to evaluate the diagnostic values of BPD, HC, AC, FL and EFW discordances in the third trimester for discordant growth. In the early third trimester, the areas under the curve (AUC) of EFW discordance was 0.822, and it was respectively compared with the AUCs of the other four parameters, and no significant differences were found (*P* > 0.05) (Table 5).

**Table 5:** Diagnostic values of discordances of fetal growth parameters in the early third-trimester for growth discordance^a^

Parameter	Early third-trimester									
	AUC	***P***	95% CI	Youden index	Cut-off (%)	Sensitivity (%)	Specificity (%)	PPV (%)	NPV (%)	+LR
BPD	0.688	0.004	0.62–0.75	0.34	4.60	71.43	62.50	65.57	68.63	1.90
HC	0.672	0.086	0.56–0.77	0.39	7.07	42.86	95.83	91.13	62.65	10.28
AC	0.740	< 0.001	0.67–0.80	0.40	8.02	57.14	83.43	77.52	66.06	3.45
FL	0.780	< 0.001	0.72–0.83	0.46	5.26	66.67	79.89	76.83	70.56	3.31
EFW	0.822	< 0.001	0.76–0.87	0.58	20.36	66.67	91.30	88.46	73.26	7.66

^a^*BPD* biparietal diameter; *HC* head circumference; *AC* abdominal circumference; *FL* femur length; *EFW* estimated fetal weight; *AUC* area under the curve; *CI* confidence interval; *PPV* positive predictive value; *NPV* negative predictive value; *+LR* positive likelihood ratio

In the late third trimester, the AUC of EFW discordance was 0.759 (Table 6), and it was respectively compared with the AUCs of the other four parameters. A significant difference was found in the comparison with AC discordance (*P* = 0.018). There was no significant difference in the AUC of EFW discordance between the early third trimester and the late third trimester (*P* > 0.05).

**Table 6:** Diagnostic values of discordances of fetal growth parameters in the late third-trimester for growth discordance^a^

Parameter	Late third-trimester									
	AUC	***P***	95% CI	Youden index	Cut-off (%)	Sensitivity (%)	Specificity (%)	PPV (%)	NPV (%)	+LR
BPD	0.634	0.005	0.58–0.69	0.27	3.49	67.44	59.72	62.61	64.72	1.67
HC	0.617	0.048	0.54–0.69	0.23	5.81	38.71	84.72	71.70	58.02	2.53
AC	0.683	< 0.001	0.63–0.73	0.29	7.69	44.19	85.11	74.80	60.39	2.97
FL	0.751	< 0.001	0.70–0.80	0.43	5.97	51.16	91.84	86.24	65.28	6.27
EFW	0.759	< 0.001	0.71–0.80	0.43	14.37	69.77	73.50	72.47	70.86	2.63

^a^*BPD* biparietal diameter; *HC* head circumference; *AC* abdominal circumference; *FL* femur length; *EFW* estimated fetal weight; *AUC* area under the curve; *CI* confidence interval; *PPV* positive predictive value; *NPV* negative predictive value; *+LR* positive likelihood ratio

## Discussion

This study applied two different assessment strategies for data analysis. On one hand, we performed the evaluation of the predictive values of several parameters in a certain gestational duration, for example, CRL, NT and DVP in early pregnancy, and tried to select the parameter with the best predictive value; on the other hand, from the mid-pregnancy to the end of pregnancy, the parameters used to evaluate fetal growth were all BPD, HC, AC, FL and EFW, but even for the same outcome, the predictive value of each parameter was expected to be different in different gestational durations. The two different assessment approaches would lead diverse thoughts into twin growth evaluation.

Based on the above research strategy, this study found that the discordances of CRL, NT and DVP in the early pregnancy did not significantly increase in the twins those finally developed discordant growth, which was not sufficient to suggest their associations. In the mid-pregnancy, although the discordance of HC increased in twins with discordant growth, it was found that the discordance of HC was not significantly related to the occurrence of growth discordance when potential confounding factors were taken into considerations. Compared with the early pregnancy and mid-pregnancy, the growth parameters in late pregnancy showed predictive values; notably, when the discordance of EFW was ≥15–20%, the diagnostic value was inclined to be better.

From the latter perspective, the predictive values of the fetal growth parameters in late pregnancy were better than those in early pregnancy and mid-pregnancy; from the former perspective, the predictive value of EFW discordance was better than that of BPD, HC, AC and FL, and the predictive values of fetal growth parameters were better than that of DVP. We speculated that, the unbalanced growth in early pregnancy may not equate to a greater possibility of developing growth discordance in the future. Normal twins may also show growth imbalance in the early stage, and growth imbalance occurring in the early pregnancy and mid-pregnancy may not prompt a frequent monitoring. For the assessment of growth discordance, the main clinical reference should be based on growth parameters, which were the most important parameters in growth assessment and directly reflected the fetal growth and development.

Interestingly, some researchers also investigated the relationship between mild-medium twin discordance and adverse perinatal outcomes in the early stage. For example, CRL discordance ≥11% was associated with increased risk of fetal loss at < 20 weeks’ gestation [13]; CRL discordance ≥10% was associated with increased risk of birth weight discordance of ≥20% in dichorionic and monochorionic twins and preterm birth at < 34 weeks in dichorionic twins [14]. So small twin growth discordance in early pregnancy may be associated with adverse outcomes that may not be ignored. Litwinska E, et al. [15] Suggested that in dichorionic twins with CRL discordance ≥15%, a scan should be offered at 16 weeks’ gestation to examine whether the finding from the 12 weeks scan that the fetuses were anatomically normal was true and to monitor fetal growth (although the ISUOG policy was that ultrasound examination should be done every 4 weeks from 20 weeks after 11–13 weeks). Currently, this remains to be further investigated.

A previous study also noted that the longitudinal assessment of fetal development was more important and was more conducive to the timely detection of high-risk populations with discordant growth [16]. EFW and AC discordance in the mid-pregnancy were thought to have poor predictive values for weight discordance ≥25% [17]. In a study that spanned middle to late pregnancy (20–36 weeks), it was found that compared to the AC ratio (AC for larger twin/AC for smaller twin), the EFW discordance suggested by the last ultrasound before delivery (the median interval between the ultrasound week and delivery week was 8 days) had the best predictive value for a weight discordance≥20%. If the prenatal ultrasound did not suggest significant growth discordance, then for the vast majority of the cases, there would be no significant discordance in birth weight [8]. Other researchers again proved that EFW discordance was the optimal parameter for predicting a weight discordance ≥18%, and its predictive ability was inclined to be better from 24 to 36 gestational weeks [18]. These results also ascertained that the assessment of growth discordance should mainly refer to the growth parameters in late pregnancy.

It should be noted that in this study, the AUC of EFW discordance in the early third trimester was 0.822, the cut-off value was approximately 20%, and the sensitivity and specificity were 66.67 and 91.30%, respectively, and positive predictive value (PPV) and negative predictive value (NPV) were 88.46 and 73.26%, respectively. Similarly, ten previous studies in the literature suggested that the predictive value of EFW discordance (20% or 25%) had sensitivity that ranged from approximately 23–93%, specificity that ranged from approximately 80–98%, PPV that ranged from approximately 33–82% and NPV that ranged from approximately 86–97% [19]; our findings of sensitivity, specificity and PPV were consistent with these results.

The realization of the clinical practice utility is the significance of our jobs. If we could perform the ultrasound evaluation with appropriate parameters or in an appropriate gestational week, twins who were at a high risk in developing growth discordance would be found earlier, paid more attention, monitored closely and terminated at an appropriate week, thus better perinatal outcomes could be expected. However, further researches are required to determine its clinical feasibility.

The strengths of our study were the research design. In the study, the two assessment approaches were carried out at the same time to evaluate the prediction value of ultrasound parameters for twin discordant growth, and the assessment longitudinally with gestational week seemed to be more preferable to establish better clinical significance and potential applications in the future.

The main limitation of our study was the retrospective cohort study with an inherent risk of bias. And it was not a multi-center study with an insufficient sample size, thus, some results were required further confirmation in a large population study. To investigate the changing rules of growth parameter discordance with gestational week in dichorionic and monochorionic twins is the job demanding prompt solution. Besides, “birth weight discordance” was the main outcome in the study, we haven’t paid attention to growth discordant twins with small for gestational age, which may be another research point in the future.

## Conclusion

In conclusion, two different assessment approaches were suggested and adopted for the investigation to predict twin growth discordance in the current study. Twin growth should be assessed longitudinally and dynamically. Normal twins may show growth imbalance in the early stage. The discordance of EFW in late pregnancy may be a useful indicator for a growth discordance of more than 25%, and its clinical practice utility is depending on further investigations.

## Data Availability

The data that support the findings of this study are available from the corresponding author ZH upon reasonable request.

## Abbreviations

AC: Abdominal circumference
AUC: The areas under the curve
BPD: Biparietal diameter
CI: Confidence interval
CRL: Crown-rump length
DVP: Deepest volume pocket
EFW: Estimated fetal weight
FL: Femur length
HC: Head circumference
LMP: Last menstruation period
NPV: Negative predictive value
NT: Nuchal translucency
OR: Odds ratio
PPV: Positive predictive value
ROC: Receiver operating characteristic curve
SGA: Small for gestational age
